# A Novel *In Vitro* Tool to Study Cyst Nematode Chemotaxis

**DOI:** 10.3389/fpls.2020.01024

**Published:** 2020-07-14

**Authors:** Yohana Laloum, Bruno Ngala, Mélina Ianszen, Isabelle Boulogne, Carole Plasson, Sylvain Fournet, Maxime Gotté, Éric Nguema-Ona, Anne-Claire Le Roux, Virginie Gobert, Azeddine Driouich, Maïté Vicré

**Affiliations:** ^1^Normandie Université, SFR Normandie Végétal FED 4277, Université de Rouen, Laboratoire Glyco-MEV EA4358, Mont Saint-Aignan, France; ^2^FN3PT/inov3PT, Recherche, Développement, Innovation des Producteurs de Plants de Pomme de terre, Achicourt, France; ^3^IGEPP, INRAE, Institut Agro, Univ Rennes, Le Rheu, France; ^4^Centre Mondial de l'Innovation Roullier, Laboratoire de Nutrition Végétale, Pôle Stress Biotique, Saint Malo, France

**Keywords:** attraction, cyst nematode, *Globodera pallida*, root border cells, root exudates, two-choice *in vitro* assay, potato (*Solanum tuberosum* L.), tomato (*Solanum lycopersicum* L.)

## Abstract

This study presents a novel three-dimensional (3D) tool “3D *in vitro* choice” for chemotaxis assays with cyst nematodes. The original 3D *in vitro* choice was customized through digital printing. Freshly hatched second stage juveniles (J2s) of the cyst nematode *Globodera pallida* were used as the nematode model to illustrate chemo-orientation behavior in the 3D system. The efficiency and reliability of the 3D *in vitro* choice were validated with 2% Phytagel as navigation medium, in three biological assays and using tomato root exudates or potato root border cells and their associated mucilage as a positive attractant as compared with water. For each biological assay, J2s were hatched from the same population of a single generation glasshouse-cultured cysts. This novel easy to use and low-cost 3d-device could be a useful replacement to Petri dishes assays in nematode behavioral studies due to the ease of deposition of nematodes and test substances, coupled with its distinctive zones that allow for precision in choice making by the nematodes.

## Introduction

Plant-parasitic nematodes are major agricultural pathogens causing severe damage to crops leading to considerable economic losses (Nicol et al., 2011). Cyst nematodes are among the most economically damaging plant-parasitic nematodes. The cyst nematode *Globodera pallida* is an obligatory parasite specific to solanaceous crops including potato (*Solanum tuberosum* L.) tomato (*Solanum lycopersicum* L.) and eggplant (*Solanum melongena* L.) (Sullivan et al., 2007) and is one of the most devastating quarantine pests of potato crop (Jones et al., 2013). This plant parasitic nematode causes potato tuber losses of up to 9% in Europe every year (Moens et al., 2018). Preventive measures, host resistance, prolonged rotation periods are normally employed in integrated control to reduce nematode population densities in infested soils, although their effectiveness are sometimes limited. Chemicals such as soil fumigants and granular nematicides have been effective in controlling *G. pallida* to maintain the agricultural productivity (Dandurand et al., 2019). However, political pressure, environmental and human health concerns have led to deregistration or restricted applications within the EU by the European Legislation (Reg. EC 1107/2009; 459/2010 and 293/2013). Consequently, alternative solutions to conventional nematicides with little or no environmental impact are of urgent need to replace the conventional agricultural products. The development of new crop protection strategies such as the utilization of biocontrol agents (nematophagous fungi or antagonistic bacteria, Adam et al., 2014; Juan et al., 2015; Mei et al., 2018), or bio-products (Askary, 2015; Eissa and Abd-Elgawad, 2015) as potential biocidal or repellent agents could be useful to reduce plant parasitic cyst nematodes. Application of biological agents or molecules to control cyst nematode populations requires an in-depth understanding of their life cycle.

The life cycle of *G. pallida* includes egg, cyst, juvenile and adult stages. In response to hatching factors present in root exudates, *G. pallida* second-stage juveniles (J2s) hatch from the encysted eggs (Perry, 2002). Upon hatch, chemo-perception of root diffusates by J2s induce attraction towards the host root followed by infection (Curtis, 2008). According to Riga (2004) chemo-orientation is « *the ability to compare the gradient of an attractant or repellent compound to determine if the organism will orient to and move up or down the gradient* ». Recognition of signals from root chemical compounds by the sensory organs of J2 is essential to guaranteed orientation, host location and plant infection (Mclaren, 1976; Rasmann et al., 2012).

The *perception* of chemical gradients by free-living stages of plant parasitic nematodes usually occurs in three-dimensions (3D). Most attraction assays have employed either two-dimensional (2D) gradients on agar (Rende et al., 1982; Riddle and Bird, 1985; Le Saux and Quénéhervé, 2002; Spiegel et al., 2003; Wuyts et al., 2006; Hosoi et al., 2017; Warnock et al., 2017), or 3D gradients in Pluronic F-127 (Wang et al., 2009; Ngala et al., 2016; Fleming et al., 2017; Čepulytė et al., 2018), or a combination of both media (Shivakumara et al., 2018), all on Petri dishes. These Petri dish systems have been successful, but not without the constrains of the demarcation zone for test substances and the target organism. In most of the assays on Petri dish systems, inoculation of test organisms has been put in the middle of the plate while the test substances were placed at the edges of the plate or vice versa. The challenges have amongst others, involved quantification for selective orientation toward or from a particular gradient by the organism. There has therefore been an apparent need for 3D systems with distinctive zones for both test substances and the targeted organism to enhance chemo-perception studies in nematology and related disciplines.

In a bid to optimize the three dimensional system for chemotaxis assays for a better understanding of the behavior of infective juveniles towards or away from chemical signals, we hereby present a novel 3D system “3D *in-vitro* choice” for chemotaxis bioassays. The block for the “3D *in vitro* choice” was digitally printed with transparent resin to allow for stereomicroscopic observations. More precisely, the 3D block was created to offer a two-choice preference to freshly hatched J2s. As an alternative to the commonly used agar or Pluronic F-127, 2% Phytagel (Kessin et al., 1996) adapted perfectly well as a transparent medium for the 3D system as it allows for three-dimensional movement for short distance orientation of J2s.

## Materials and Methods

### Nematode Cultures and Root Exudates

The population of *G. pallida* used in this study was from a single generation cultured on the susceptible potato *S. tuberosum* L. cv. Désirée (Solanaceae) in the glasshouse during the spring season of 2017. Collected cysts were stored at 4°C for at least six months to break facultative dormancy. For each chemotaxis bioassays, 240 cysts were incubated in potato root exudates at 30 mg C g^−1^ of dry mass (Ngala et al., unpublished) in the dark at 20°C over a seven-day period. Exudates were renewed on the 4th day, while J2s were collected at days sixth and seventh, rinsed on 10-µm mesh with mineral water before immediate usage for the chemotaxis bioassays following each collection.

### Production of Roots Exudates

The tomato root exudates used in the chemotaxis assays were kindlys provided by the nematology laboratory of IGEPP, INRAE, Le Rheu, France, and was produced from *S. lycopersicum* L. cv. St. Pierre (Solanaceae). The dry matter and carbon content of the root exudates were analyzed by *Centre Mondial de l'Innovation* (CMI) Roullier, St. Malo, France. The exudates were kept frozen at −25°C in 50 ml falcon tubes until required for the bioassays.

### Obtention of Potato Root Border Cells and Mucilage

Border cells and their associated mucilage were collected from potato tuber (*S. tuberosum* L. cv Désirée) kindly provided by RD3PT, Achicourt, France. In order to produce potato germs, potato tubers were grown in a culture chamber at 20°C in the dark during 4 weeks. Potato germs were then placed in a hydroponic solution during 4 weeks and grown in a culture chamber at 23°C in dark condition. Roots were briefly rinsed and immersed in 3 ml of water. Border cells were harvested from the root tip by gentle agitation during 15 to 30 s in water and immediately used for chemotaxis assay. The border cells fraction also contains root exudate molecules. Root exudates contribute many types of macromolecules including polysaccharides and glycoproteins that are produced by root border cells known to detach from the root tip when this is placed in water (Driouich et al., 2019). Therefore, potato border cells and their secretions are part of root exudates.

### Description of the 3D *In Vitro* Choice

**Figure 1:**
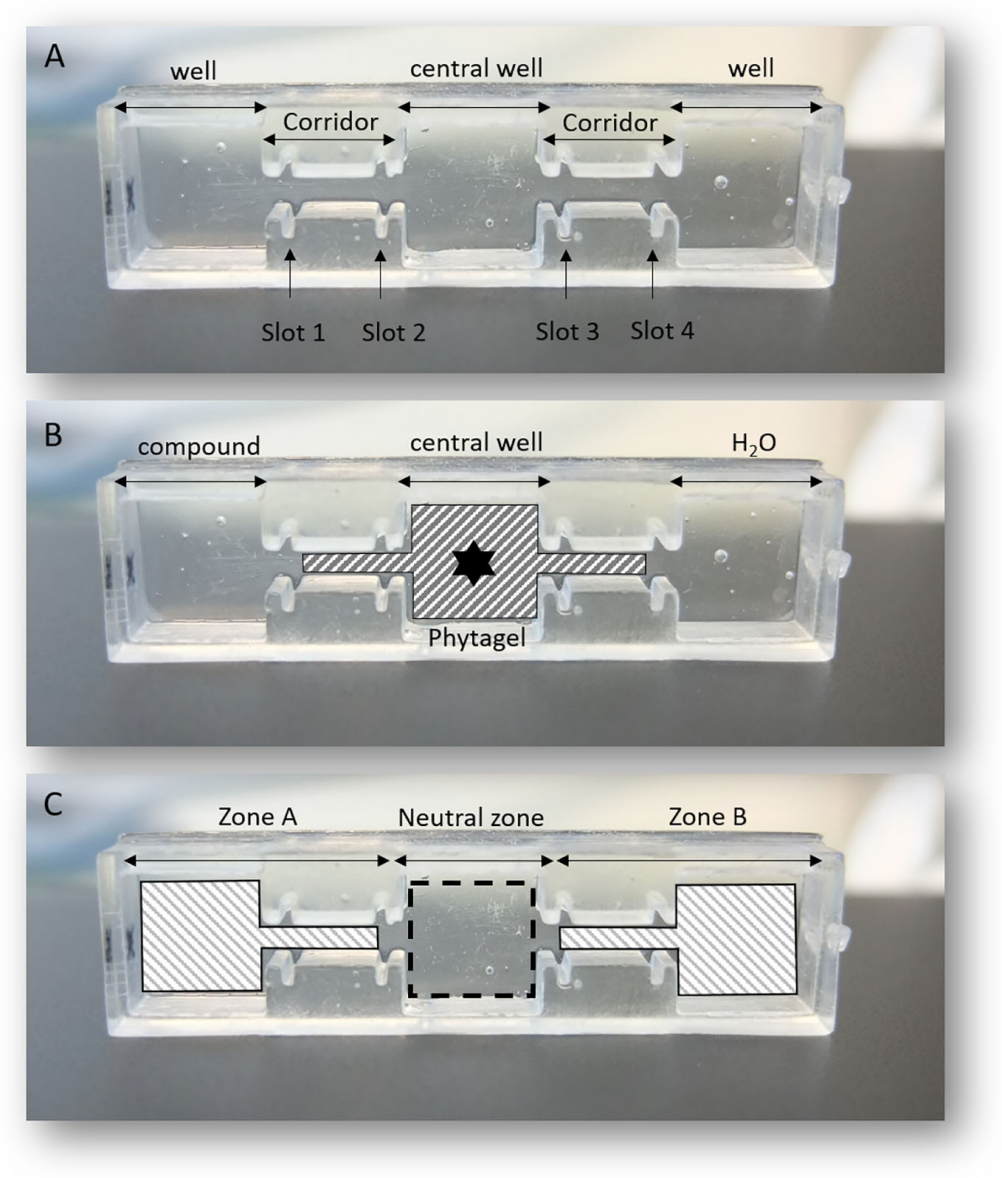
An illustration of the 3D *in vitro* choice printed by Digital Light Processing printer; **(A)** the partitioning of the different compartments; **(B)** illustrates the experimental designs involving the addition of 2% Phytagel, addition of test solutions (compound or water) and the point of J2s inoculation (black star); **(C)** illustrates different zones in which J2s were counted.

The 3D device (**Figure 1**) was printed with a digital light processing printer (Wanhao Duplicator 7) by Normandiy 3D (Notre-Dame-de-Bondeville, Normandie, France). The 3D *in vitro* choice has been printed in transparent photo-reactive resin which solidifies layer by layer to form the object by DLP (Digital Light Processing) printing. The setup parameters of the machine used to create the device can be obtained from the Normandiy 3D company. The organization of the different compartments of the 3D *in vitro* choice (**Figure 1A**) includes a central well connected to two external wells by two narrow channels (corridors). The different sections are separated from each other by the insertion of movable plastic walls into the slots (slot-1 to slot-4). The external wells are equidistant from the central well. Each well has the same dimensions (1.0 × 1.0 × 0.5 cm^−3^). **Figure 1B** illustrates the experimental design for the 3D *in vitro* choice to test attraction or repulsion of test compounds. Phytagel (2%) is spread to occupy the central well and the two channels interconnecting the external wells. Aliquots of 300 µl of test solutions or water are each added into their respective outer wells. The black star at the center of the central well indicates the point of J2s inoculation. Inoculated J2s are allowed in the 3D *in vitro* choice plate over 24 h in dark for sensory perception and navigation. An important aspect of the 3D *in vitro* choice is based on the fact that J2s, which choose to enter into the adjacent wells, are trapped in the liquid solution, thus, a backward navigation into the Phytagel at this point is impossible. **Figure 1C** illustrates the areas under which J2s were scored to represent attraction or repulsion (zone A), no choice (neutral zone) or control (zone B).

### Preparation of the 3D *In Vitro* Choice

The first step into the setup off the 3D device involved separating the external wells from the connective channels by inserting the movable plastic walls into slots 1 and 4 (**Figure 1A**) to prevent the flow of phytagel into adjacent wells. An aliquot of 500 μl of 2% Phytagel (Sigma-Aldrich), dissolved in deionized water, was poured into the central well and the two adjacent channels (corridors). The gel was then allowed to cool down for 20 to 30 min at room temperature and pressure (r.t.p.), before the withdrawal of the movable walls (**Figure 1B**). Phytagel at 2% enabled 3D navigation of infective juveniles within the central well. Aliquots of 300 μl of the test solution or mineral water were then added into their respective outer wells (**Figure 1B**).

### Validation of the Effectiveness of the 3D *In Vitro* Choice

To validate the effectiveness and reliability of the 3D *in vitro* choice, three experimental conditions were tested which involved a neutral condition and a positive control. The neutral condition aimed at validating the random navigation of J2s on the 3D *in vitro* choice in the absence of any attractant or repellent compound. Under that condition, both adjacent wells contained 300 μl of mineral water.

Unlike for the neutral control, the positive control aimed at validating the effectiveness of sensory-driven orientation of J2s in the 3D *in vitro* choice. Attractiveness toward root exudates of tomato and potato root border cells fraction was tested against mineral water. As such, 300 μl of tomato root exudates (0.01 mg of carbon g^−1^ of dry mass) or 300 µl solution of potato root border cells solution and associated mucilage were added into the left well, while 300 μl of mineral water were added into the opposite well.

For each set of experiments, approximately 200 ± 40 freshly hatched *G. pallida* J2s were pipetted in 5 μl of mineral water and deposited onto the Phytagel in the central well. Following inoculation of J2s, the bioassays were incubated at 20°C in the dark for 24 h, before the nematodes present in each well/channel were counted under a stereomicroscope (Olympus SZX2-ILLT). Results have been achieved through three independent biological assays with ten technical replicates for each one.

### Determination of J2s Chemotaxis Index

Attractiveness or repulsion effects of the test compound were estimated by quantifying the distribution of J2s which made a choice. Only the J2s which moved out of the neutral zone (**Figure 1C**) were counted for the chemotaxis index. For each experiment, a chemotaxis index (CI) was calculated using Equation (1) based on previous studies (Margie et al., 2013; Fleming et al., 2017; Warnock et al., 2017; Shivakumara et al., 2018)

(1) $$\text{CI}=\frac{\text{A}-\text{B}}{\left(\text{A}+\text{B}\right)}\;\text{... ... ...}$$

Where:

CI = chemotaxis index
A = the number of J2s within zone A (test compound)
B = the number of J2s within zone B (control)

A positive score (+) corresponds to attraction whereas a negative score (−) corresponds to repulsion. Chemotaxis index score closer to ±1 indicates the strength of the attraction or repulsion respectively, whereas CI scores between ±0.2 indicates a lack of response to the test compound.

### Determination of the Mean Percentage of Migration of J2s

The percentage of migration represented by the total number of J2s which made a choice was calculated as an average proportion of the inoculated J2s using Equation (2).

(2) $$Migration\;\left(\%\right)=\left[\frac{A+B}{\left(A+B+T\right)}\right]\times 100\;\text{... ... ...}$$

Where:

*T* = number of J2s in the neutral zone (central well) after 24 h.

### Statistical Analyses

Statistical analyses were generated using GraphPad Prism version 7. Significant chemotaxis response of *G. pallida* J2s to tomato root exudates and potato root border cells fraction as compared to controls (water) was determined according to Wilcoxon nonparametric test (5% level of significance).

## Results and Discussion

### Random Movement of *G. pallida* J2s in the 3D *In Vitro* Choice in Neutral Conditions

**Figure 2:**
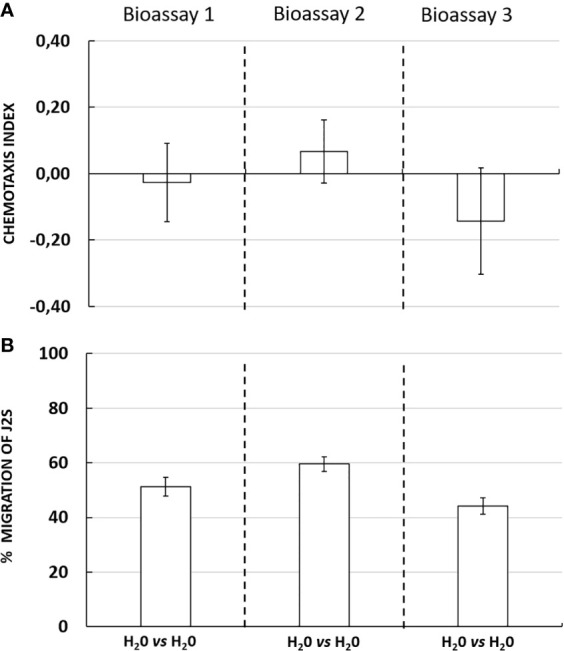
**(A)** Chemotaxis response of *Globodera pallida* J2s in neutral conditions in three independent bioassays with ten technical replicates each. Chemotaxis bioassays show attraction (positive index) or repulsion (negative index). **(B)** Percentage migration of *Globodera pallida* J2s. Error bars indicate the standard error of means (SEM) for ten replicates (*n = 10*).

According to the result of the three chemotaxis bioassays, chemotaxis index corresponding to the neutral condition (water *vs* water) were −0.03 ± 0.12; 0.07 ± 0.10 and −0.14 ± 0.16, respectively (**Figure 2A**). No significant differences were observed for each assay. The percentage migration of J2s in the tree bioassays was 51% ± 3; 60% ± 3 and 44% ± 3, respectively (**Figure 2B**). The data indicate a homogeneous distribution of J2s on the 3D *in vitro* choice in neutral conditions confirming that in the absence of any attractant or repellent solution, J2s showed an unselective orientation behavior. These observations also confirmed that the behavior and distribution of *G. pallida* J2s were not affected by the design or the material composition of the 3D *in vitro* system itself.

### Validation of the Effectiveness and Reliability of the Novel 3D System “3D *In Vitro* Choice”

**Figure 3:**
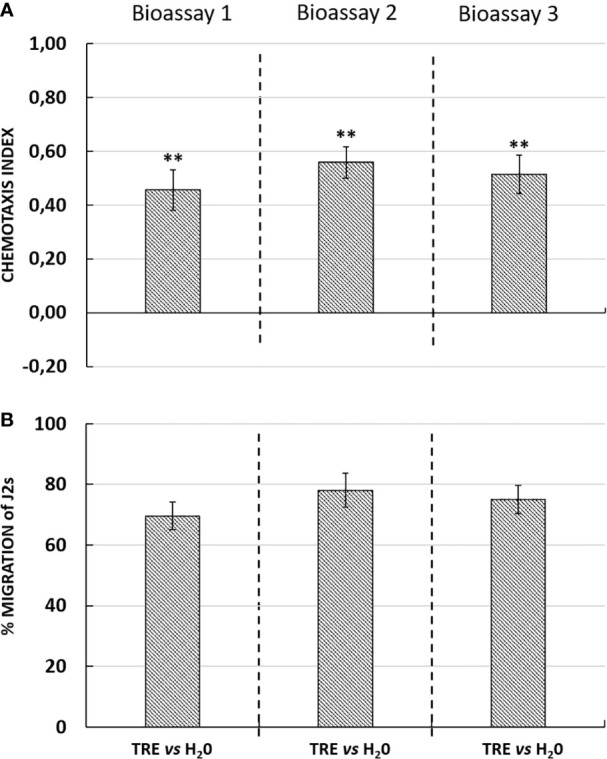
**(A)** Chemotaxis response of *Globodera pallida* J2s to tomato root exudates (TRE) in three independent bioassays with ten technical replicates each. Chemotaxis bioassays show attraction (positive index) or repulsion (negative index). Statistical significance between TRE and H_2_O is indicated by stars (**) according to Wilcoxon nonparametric test (5% level of significance) (**p <0.01). **(B)** Percentage of migration of *Globodera pallida* J2s in three independent bioassays. Error bars indicate the standard error of the means (SEM) for ten replicates (*n* = 10).

**Figure 4:**
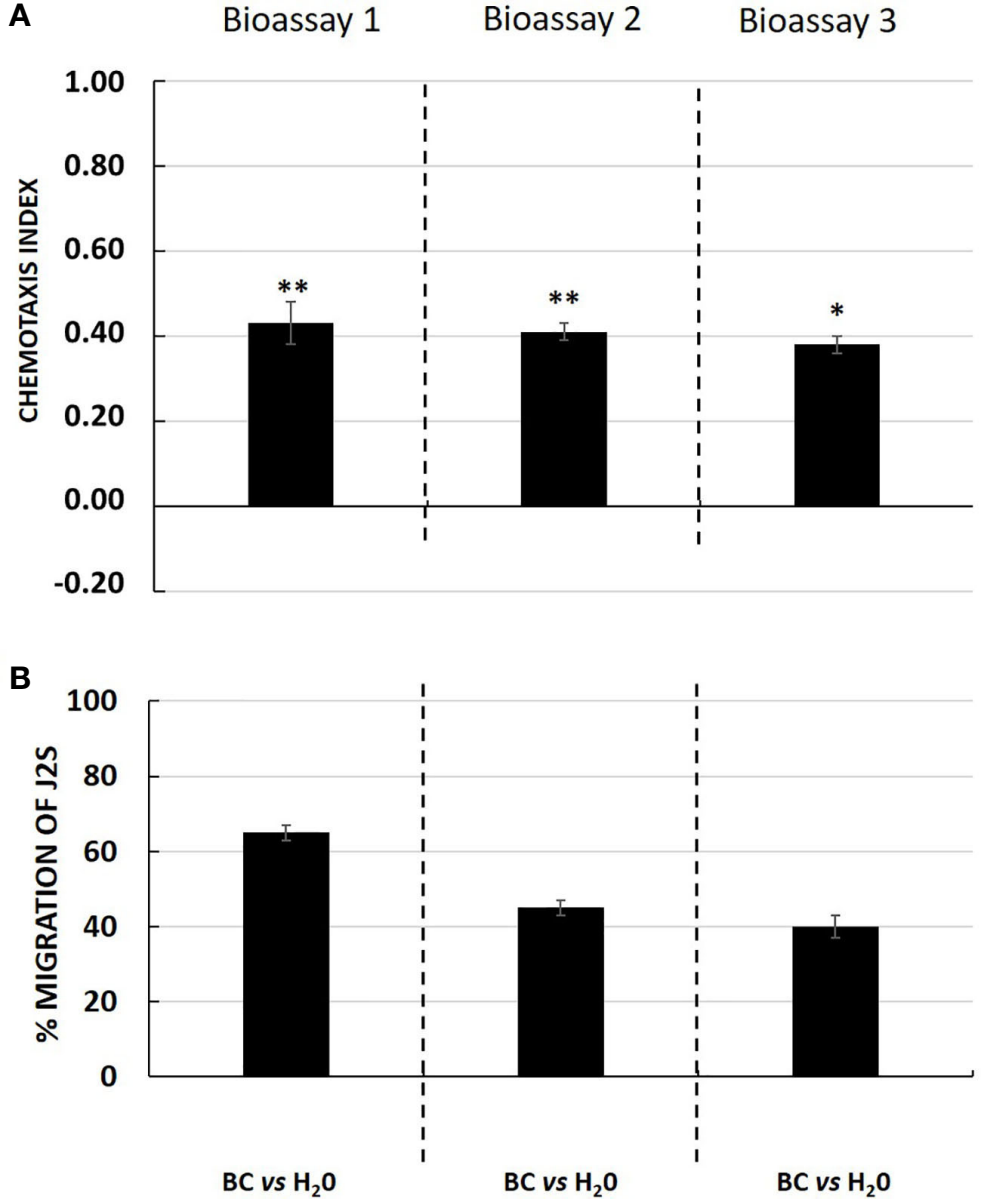
**(A)** Chemotaxis response of *Globodera pallida* J2s to potato root border cells and its associated mucilage fraction (BC) in three independent bioassays with ten technical replicates each. Chemotaxis bioassays show attraction (positive index) or repulsion (negative index). Statistical significance between BC and H_2_O is indicated by stars (*; **) according to Wilcoxon nonparametric test (5% level of significance) (*p <0.05; **p <0.01). **(B)** Percentage of migration of *Globodera pallida* J2s in three independent bioassays. Error bars indicate the standard error of the means (SEM) for ten replicates (*n* = 10).

Interestingly, in the presence of an attractant solution, the majority of J2s moved out of the neutral zone towards the attractive zone, illustrating the ability of tomato root exudates to induce J2s decision-making as compared with water (control well) (**Figure 3A**). Similarly, root border cells and mucilage from potato were also shown to attract *G. pallida* J2s (**Figure 4A**). Root border cells are cells that detached from the root tip when placed in water and that are released in the rhizosphere together with the surrounding mucilage. Mucilage and root border cells formed the “Root Extracellular Trap or RET” involved in root protection against soil-borne pathogens such as nematodes (Hawes et al., 2000; Driouich et al., 2013; Driouich et al., 2019). The results indicated that both tomato root exudates and potato root border cells fraction induced a significant positive chemotaxis index of 0.46 ± 0.07; 0.56 ± 0.06 and 0.51 ± 0.07 and 0.43 ± 0.05; 0.41 ± 0.02 and 0.38 ± 0.02 in the three bioassays respectively (**Figures 3A** and **4A**). For each bioassay, statistical tests indicated that *G. pallida* J2s were significantly attracted towards tomato root exudates as compared with the control (water). The percentage migration of J2s in the three bioassays for tomato root exudates was 70% ± 4; 78% ± 6 and 75% ± 5 and for potato root border cells fraction was 65% ± 2; 45% ± 2; 40% ± 3 respectively (**Figures 3B** and **4B**). Most, if not all the J2s observed in the neutral zone (zone of inoculation) after 24 h remained immobile or on a straight posture as compared with J2s found in the channels and in the well containing tomato root exudates, which were all actively moving. These results confirmed the attractiveness of *G. pallida* J2s towards tomato root exudates as previously demonstrated (Devine and Jones, 2003). In addition, in this study, tomato root exudates were shown to induce a minimum chemotaxis index of 0.4 as previously observed in a Petri dish system (Warnock et al., 2017).

Unlike agar and Pluronic F-127 gel, 2% Phytagel offered a transparent medium with relative ease of mobility by J2s that enabled observation and scoring under a stereomicroscope. The distinctive wells made it much easier to quantify selective behavior of J2s unlike the Petri dish systems in which the demarcation of zones is not easily distinguishable. Moreover, the relatively short distances between adjacent wells made dissemination of attractant or repellent molecules easier for the nematode to perceive, thus enabling selective orientation. An additional advantage of the 3D *in vitro* choice is the cost, which is very affordable and well adapted to stereomicroscopic observations, making it possible to assess a high number of samples within a short time frame considering the rate of lipid reserves utilisation by J2s at r.t.p.

## Conclusion

In conclusion, the 3D *in vitro* choice represents a novel, inexpensive and reliable tool that quantitatively measures attraction of nematodes in response to biological control agents/molecules. The cyst nematode *G. pallida* was used as the nematode model to illustrate chemo-orientation behavior in the 3D system. However, this can also be applied to other biological organisms including root-knot nematodes, bacteria, oomycetes or fungi and will promote studying the complex network of *G. pallida* interactions during the host finding process.

## Data Availability Statement

The raw data supporting the conclusions of this article will be made available by the authors, without undue reservation.

## Author Contributions

YL designed and performed the experiments. BN, SF, IB, CP and MV contributed to designing the experiments and analyzing the data. YL wrote the first version of the manuscript with the assistance of BN and SF. MV and AD supervised the research. BN, SF, AD, IB, ÉN-O and MV revised the manuscript. All authors contributed to the article and approved the submitted version.

## Conflict of Interest

The authors declare that the research was conducted in the absence of any commercial or financial relationships that could be construed as a potential conflict of interest.
